# Molecular evidence for asymmetric hybridization in three closely related sympatric species

**DOI:** 10.1093/aobpla/ply011

**Published:** 2018-02-09

**Authors:** Ning-Ning Zhang, Jiao-Jun Yu, Yue-Hua Wang, Xun Gong

**Affiliations:** 1 Plant Science Institute, School of Life Sciences, Yunnan University, 650201 Kunming, Yunnan, China; 2 Key Laboratory for Plant Diversity and Biogeography of East Asia, Kunming Institute of Botany, Chinese Academy of Sciences, 650201 Kunming, Yunnan, China; 3 University of Chinese Academy of Sciences, 100049 Beijing, China

**Keywords:** Asymmetric hybridization, chloroplast DNA, F_1_ generation, *Ligularia*, nuclear loci, two hybridization groups

## Abstract

Natural hybridization is common in plants and results in different evolutionary consequences to hybridizing species. Pre- and post-zygotic reproductive isolating barriers can impede hybridization between closely related species to maintain their species integrity. In Northwest Yunnan, three *Ligularia* species (*Ligularia cyathiceps*, *L. duciformis* and *L. yunnanensis*) and four types of morphologically intermediate individuals were discovered growing together in an area subject to human disturbance. In this study, we used three low-copy nuclear loci to test the natural hybridization hypothesis and the hybridization direction was ascertained by three chloroplast DNA fragments. The results indicated there were two hybridization groups at the study site, *L. cyathiceps* × *L. duciformis* and *L. duciformis* × *L. yunnanensis*, and two types of morphologically intermediate individuals were produced by *L. cyathiceps* and *L. duciformis*, and another two types were produced by *L. duciformis* and *L. yunnanensis*, while no hybrids between *L. cyathiceps* and *L. yunnanensis* were observed. Both hybridizing groups showed bidirectional but asymmetric hybridization and the factors influencing the symmetry are discussed. Most hybrids produced by the two hybridization groups seemed to be F_1_ generation. Hybrids with different morphologies within the same hybridization group showed similar genetic components. The results suggest that although human disturbance may promote natural hybridization among the three *Ligularia* species bringing them together, hybrids are limited to F_1_s and therefore species boundaries might be maintained.

## Teaser

Natural hybridization is common in plants and results in different evolutionary consequences for hybridizing species. Here, we used three low-copy nuclear loci to test the natural hybridization hypothesis among three *Ligularia* species and the hybridization direction was ascertained by three chloroplast DNA fragments. There were two hybridization groups at the study site, and they both showed bidirectional but asymmetric hybridization. Although natural hybridization occurs in three *Ligularia* species, their hybrids seemed to be limited to the F_1_ generation and therefore species boundaries might be maintained.

## Introduction

Natural hybridization is common across plants, particularly in rapidly radiating groups (Mallet 2005), and can result in both positive and negative evolutionary outcomes (Barton 2001). Hybridization can generate new taxa through homoploid or allopolyploid hybrid speciation; however, it can also reduce the species diversity by blurring species boundaries, especially if introgression occurs (Wang *et al.* 2001; Ramsey and Schemske 2002; Mallet 2007; Schneider *et al.* 2011; Beatty *et al.* 2014). Species integrity is maintained by pre- and post-zygotic reproductive isolating barriers preventing hybridization (Dickinson *et al.* 2012). However, human disturbance is regarded as an important promoter of hybridization (Anderson 1948; Bleeker and Hurka 2001), and previously isolated species may come into contact and hybridize due to human alterations to the environment (Rhymer and Simberloff 1996; Seehausen *et al.* 2008; Crispo *et al.* 2011; Bohling *et al.* 2016). Human disturbance has been proved to increase hybridization rates in some plants, such as breaking geographical barriers and promoting biological invasions (Thompson *et al.* 2010), changing fire regimes (Ortego *et al.* 2017) or even changing biological attributes as phenology (Lamont *et al.* 2003). When hybridization happens, the direction of hybridization is affected by both pre- and post-zygotic barriers and asymmetric hybridization frequently occurs in plants as a result of differences in the strength of reproductive barriers between hybridizing species (Bacilieri *et al.* 1996; Arnold 1997; Ma *et al.* 2014; Zhang *et al.* 2016).

*Ligularia*, a highly diversified genus belonging to Senecioneae (Asteraceae), is comprised of about 140 species distributing in Asia and Europe (Liu and Illarionova 2011) and its major distribution centre is located in Hengduan Mountains (Liu *et al.* 1994). Natural hybridization is frequent in *Ligularia* and natural hybrids are commonly found in areas of sympatry (Liu *et al.* 2006). Pan *et al.* (2008) firstly reported *Ligularia* × *maoniushanensis* was a natural hybrid produced by *Ligularia paradoxa* and *Ligularia duciformis* in Yunnan, China. Yu *et al.* (2011,2014a, b) proved natural hybridization of *Ligularia nelumbifolia* and *Ligularia subspicata*, *Ligularia vellerea* and *L. subspicata* and between *Ligularia cymbulifera* and *Ligularia tongolensis* by using the internal transcribed spacer (ITS) region of the nuclear ribosomal DNA and chloroplast DNA (cpDNA) fragments. Moreover, studies on chemical compounds combined with nuclear ribosome ITS sequence also confirmed natural hybridization of *L. nelumbifolia* and *L. subspicata*, and of *L. cymbulifera* and *L. tongolensis* (Hanai *et al.* 2012; Shimizu *et al.* 2016). In all the cases described above, natural hybridization usually forms complex hybrid swarms and gene introgression between parental species, which may blur species boundaries between hybridizing species.

During the field investigation in Tianchi (Shangri-La, Yunnan), a place severely disturbed by farming, deforestation and tourism, three *Ligularia* species (*Ligularia cyathiceps*, *L. duciformis* and *L. yunnanensis*) were found growing together and four types of morphologically intermediate individuals (Type A, B, C and D) were discovered. According to morphological distinction, it is assumed that there are two hybridization groups, i.e. Type A and B individuals are presumed to be hybrids of *L*. *cyathiceps* and *L*. *duciformis*, while Type C and D individuals are supposed to be hybrids of *L*. *duciformis* and *L. yunnanensis*. In spite of frequently reported studies on natural hybridization of *Ligularia* in recent years, there is no report on the complicated relationships at the morphologically diverse hybrid zone described above involving three putative parents.

Natural hybrids can show various morphological characteristics, such as parental-like, intermediate or novel traits, and morphological evidence alone is inadequate in the identification of hybrids (Schneider *et al.* 2011). Molecular techniques can provide more powerful evidence for natural hybridization, and low-copy nuclear genes have been proved to be efficient in solving problems of natural hybridization (Zhang *et al.* 2013; Fan *et al.* 2014; Liao *et al.* 2015). Particularly, the utility of nuclear genes in previous studies is limited to nuclear ribosome ITS region, which have showed disadvantages such as not always tracking both parents’ genomes in hybrids (Jupe and Zimmer 1993) and amplifying pseudogenes or fungal ITS spacers (Buckler and Holtsford 1996; Muir *et al.* 2001; Song *et al.* 2012). In this study, three low-copy nuclear loci (A12, B14 and D30) and three chloroplast intergenic spacers (*psb*A–*trn*H, *trn*L–*rpl*32 and *trn*Q–5′*rps*16) were used to explore the relationships among *L. cyathiceps*, *L. duciformis*, *L. yunnanensis* and all the morphologically intermediate individuals observed in the contact zone. Our aims were to (i) identify if morphologically intermediate individuals are produced by hybridization between the three coexisting species and decouple the occurrence of two natural hybridization groups suggested by the morphologically intermediate individuals between *L. cyathiceps* and *L. duciformis* by one side and between *L. duciformis* and *L. yunnanensis* by the other side; (ii) if hybridization is confirmed, assess the direction of natural hybridization; and (iii) compare the consequences of two putative hybridization groups and their influence to three putative parental species.

## Methods

### Study species and plant sampling

The study site is located in Tianchi, Shangri-La, Yunnan, China (27°37.339′N, 99°38.151′E, 3901 m a.s.l.), where *L. cyathiceps*, *L. duciformis* and *L. duciformis* distribute sympatrically. *Ligularia cyathiceps* and *Ligularia yunnanensis* are two alpine species endemic to Northwest Yunnan with altitudes from 3000 m to 4000 m a.s.l. (Liu and Illarionova 2011). *Ligularia duciformis* distributes widely in West China and grows at altitudes varying from 1900 m to 4300 m a.s.l. (Liu and Illarionova 2011). *Ligularia duciformis* and *L. yunnanensis* belong to the series *Retuase*, section *Corymbosae*, and *L*. *cyathiceps* is a member of series *Ligularia*, section *Ligularia* (Liu 1989). These three species are all diploids with somatic chromosome number 2*n* = 58 (Pan *et al.* 2004, 2008).

**Figure 1.**
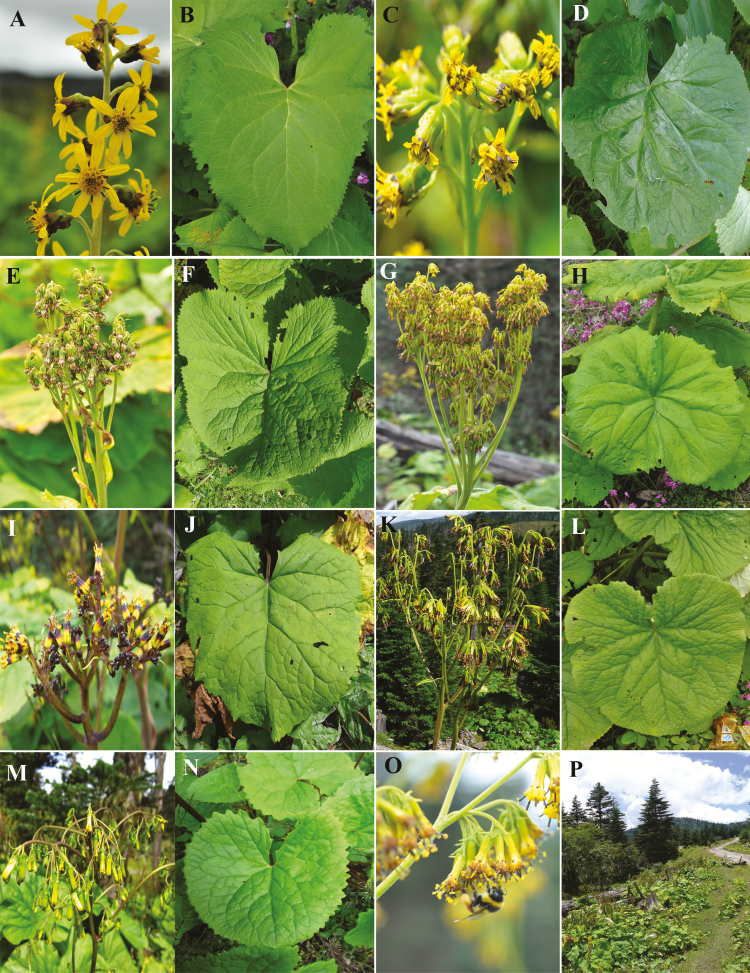
Morphological illustrations for the investigated individuals at the study site. (A and B) *Ligularia cyathiceps*; (C and D) Type A; (E and F) Type B; (G and H) *Ligularia duciformis*; (I and J) Type C; (K and L) Type D; (M and N) *Ligularia yunnanensis*; (O) the pollinator on Type D; (P) habitat.

According to the morphological descriptions in *Flora of China* (Liu and Illarionova 2011), *L. cyathiceps* and *L*. *duciformis* mainly differ in leaf size, dentate leaf margin, capitula arrangement and presence or absence of ray florets, while *L*. *duciformis* and *L. yunnanensis* have major differences in leaf size, dentate leaf margin, inflorescence branches and indumentum. The diagnostic morphological traits used to identify the species are presented in Table 1 and illustrated in Fig. 1, including the four morphologically intermediate types (Type A, B, C and D) that do not fit in the description of any of the species. Moreover, *L. cyathiceps*, *L*. *duciformis* and all putative hybrids prefer open and sunny habitats and occupy disturbed hillsides and roadsides, whereas *L. yunnanensis* likes shady and humid environment and is found at intact habitats below the canopy of trees.

A total of 148 individuals were sampled for molecular analysis. For three species *L. cyathiceps*, *L. duciformis* and *L. yunnanensis*, each of 20 individuals were collected. For four morphologically intermediate types, we sampled all the individuals with intermediate morphology and number of sampled individuals was detailed in Table 1. Healthy leaves from each individual were collected and stored in plastic bags with silica gel until DNA extraction. Voucher specimens were deposited in the Herbarium of Kunming Institute of Botany, Chinese Academy of Sciences (KUN) and voucher numbers are detailed in Table 1.

**Table 1.** Sampling details and morphologically diagnosic characteristics for *L. cyathiceps*, *L. duciformis*, *L. yunnanensis* and putative hybrids.

Taxon	No. of individuals (ID)	Voucher	Leaf size	Leaf blade margin	Inflorescence
*L. cyathiceps* (Lc)	20 (C1–20)	PG140805	8.5–13 × 10.5–22 cm	Coarsely dentate, apex rounded	Racemose, tubular florets, with several ray florets
Type A	15 (F1–15)	PG140816	Intermediate between Lc and Ld	Coarsely dentate, apex rounded	Compound corymb, tubular florets, with several ray florets
Type B	9 (T1–9)	PG140808	Intermediate between Lc and Ld	Coarsely dentate, apex rounded	Compound corymb, all tubular florets
*L. duciformis* (Ld)	20 (D1–20)	PG140813	5–16 × 7–50 cm	Denticulate, apex retuse	Compound corymb, all tubular florets, branches spreading, pubescent
Type C	30 (H1–30)	PG140814	Intermediate between Ld and Ly	Denticulate, apex retuse	Compound corymb, branches spreading relatively, shortly brown pilose
Type D	34 (S1–34)	PG140819	Intermediate between Ld and Ly	Denticulate, apex retuse	Compound corymb, branches spreading relatively, pubescent
*L. yunnanensis* (Ly)	20 (Y1–20)	PG140802	3–6.5 × 7–11 cm	Coarsely triangular-dentate, apex rounded	Corymb, branches shorter, fasciated, shortly brown pilose

### DNA extraction, PCR amplification and sequencing of DNA sequences

Total genomic DNA was extracted from dried leaves using the modified CTAB method (Doyle 1991). We amplified nearly all the universal markers applied to Asteraceae family (Chapman *et al.* 2007) and finally obtained three low-copy nuclear loci A12, B14 and D30, which could be amplified and sequenced successfully with variable sites in the investigated individuals. Primers for A12, B14 and D30 developed by Chapman *et al.* (2007) were used in the present study. For B14 and D30 loci, internal primers were designed to obtain complete sequences of some individuals. Designed primers for B14 and D30 were LB14F: 5′ AACGCRTACCTTTCCAACG 3′, LB14R: 5′ TCYGTCGCATTCTCCCTTC 3′ and LD30F: 5′ AATGTTCAGATTTTGGTTAT 3′, LD30R: 5′ CTTAGGTGAATCTGTTGC 3′, respectively. PCR conditions followed Chapman *et al.* (2007). Some individuals, especially from putative hybrids, had superimposed chromatograms at multiple sites and cloning sequencing was used to phase the haplotypes. Ligations were conducted using the pMD19-T Vector cloning kit (Takara, Dalian, China). Two to eight positive clones for each individual were selected for sequencing.

Three chloroplast intergenic spacers *psb*A–*trn*H, *trn*L–*rpl*32 and *trn*Q–5′*rps*16 were amplified using universal primers (Sang *et al.* 1997; Shaw *et al.* 2007). The PCR amplification was carried out in 20 μL reaction volume, containing 20 ng genomic DNA, 2.0 μL 10× PCR buffer, 1.0 μL MgCl_2_ (25 mM), 1.0 μL dNTPs (10 mM), 1.0 μL BSA (20 g/L), 0.2 μL *Taq* DNA polymerase (5 U/μL) (Takara, Shiga, Japan), 0.5 μL of each primer and 12.3 μL double-distilled water. PCR was conducted in a thermocycler with the following conditions: an initial 5 min denaturation at 80 °C, followed by 30 cycles of 45 s at 94 °C, 45 s annealing at 53 °C, 50 s extension at 65 °C and a final extension for 7 min at 65 °C. All PCR products were purified by electrophoresis with a 1.2 % agarose gel and then a Pearl Gel Extraction Kit (Pearl Biotech, Guangzhou, China) was used. Then, they were sequenced in both directions with the amplification primers using an ABI 3730 DNA automated sequencer with the BigDye Terminator Cycle Sequencing Ready Reaction Kit (Applied Biosystems, Foster City, CA, USA).

### Data analysis

All sequences were edited and assembled in SeqMan™ (DNASTAR, Madison, WI, USA). Multiple alignments were performed manually with Geneious Pro version 4.8.2 (Biomatters Ltd, Auckland, New Zealand). For three low-copy nuclear loci, haplotype inference was implemented with PHASE in DnaSP version 5.0 (Rozas *et al.* 2003). A congruency test for three combined cpDNA intergenic spacers showed a significant rate of homogeneity (*P* > 0.5) by PAUP*4.0b10 (Swofford 2002), indicating a high degree of homogeneity. Haplotypes of the combined chloroplast sequences were inferred using DnaSP 5.0. Haplotype network was constructed for each nuclear locus and combined cpDNA region using Network version 5.0.0.0 (Forster *et al.* 2007) with the median-joining algorithm (Bandelt *et al.* 1999). Indels were treated as single mutational events in network analysis.

## Results

### Sequence analysis of nuclear loci—A12 locus

**Figure 2.**
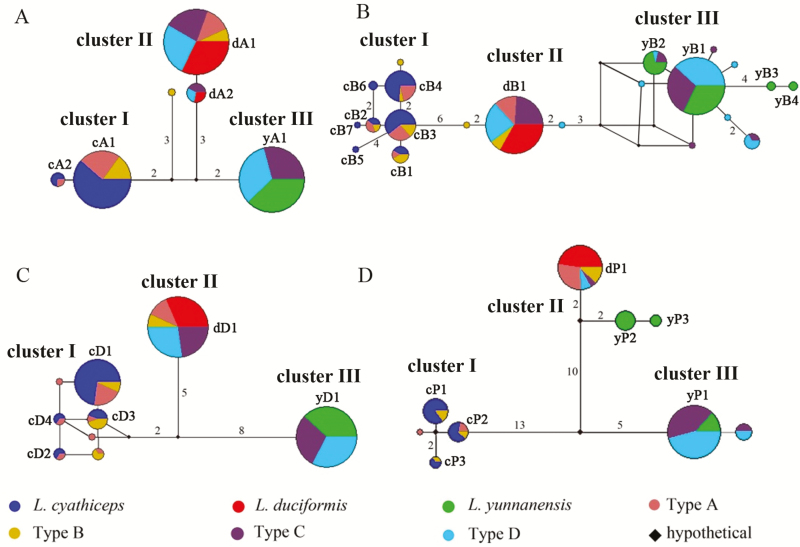
Haplotype networks for nuclear loci A12 (A), B14 (B), D30 (C) and three combined cpDNA fragments (D). Mutation steps are shown by numbers on the line, and node size is proportional to the frequency of each haplotype. For the haplotype names of three putative parental species, c, d and y represent *L*. *cyathiceps*, *L*. *duciformis* and *L. yunnanensis*, respectively.

The aligned A12 region was 257 bp in length for all the investigated individuals (for variation sites, **see****Supporting Information—Table S1**). A total of six haplotypes were derived, and low levels of haplotype polymorphism were observed in *L. cyathiceps*, *L. duciformis* and *L. yunnanensis* which had two (cA1–2), two (dA1–2) and two (yA1) haplotypes, respectively (Table 2; Fig. 2A). Haplotypes of *L. cyathiceps*, *L. duciformis* and *L. yunnanensis* generated three clusters (cluster I, II and III) in haplotype network analysis, in which *L. cyathiceps* (cluster I) and *L. duciformis* (cluster II) were separated by six nucleotide substitutions and *L. duciformis* (cluster II) and *L. yunnanensis* (cluster III) were separated by five nucleotide substitutions (Fig. 2A).

**Table 2.** Haplotypes for three *Ligularia* species and putative hybrids of three nuclear loci and combined cpDNA fragments.

Taxon	A12	B14	D30	cpDNA
*L. cyathiceps*	cA1, cA2	cB1, cB2, cB3, cB4, cB5, cB6, cB7	cD1, cD2, cD3, cD4	cP1, cP2, cP3
Type A	cA1, cA2, dA1	cB1, cB2, cB3, cB4, dB1	cD1, cD2, cD3, cD4, dD1, UN1, UN2, UN3	cP2, dP1, UN1
Type B	cA1, dA1, UN	cB1, cB2, cB3, cB4, dB1, UN1, UN2	cD1, cD3, dD1, UN2	cP1, cP2, cP3, dP1
*L. duciformis*	dA1, dA2	dB1	dD1	dP1
Type C	dA1, dA2, yA1	dB1, yB1, yB2, UN3, UN5, UN9	dD1, yD1	yP1, UN2
Type D	dA1, dA2, yA1	dB1, yB1, yB2, UN4, UN5, UN6, UN7, UN8	dD1, yD1	dP1, yP1, UN2
*L. yunnanensis*	yA1	yB1, yB2, yB3, yB4	yD1	yP1, yP2, yP3
Total haplotype number	6	21	9	9

For the putative hybrids of *L. cyathiceps* and *L. duciformis* (Type A and B), all individuals but one (T7) showed two divergent haplotypes (cA1/dA1 and cA2/dA1) originated from *L. cyathiceps* and *L. duciformis*, and the haplotype of individual T7 was a combination of two haplotypes (cA1/UN1) found in *L. cyathiceps* cluster (Table 3). For the putative hybrids of *L. duciformis* and *L. yunnanensis* (Type C and D), all individuals but six (H12, S1, S4, S8, S16 and S32) had combined haplotypes (dA1/yA1 and dA2/yA1) nested in clusters of *L. duciformis* and *L. yunnanensis* (Table 3). Individuals S8 and S16 were homozygous for a *L. duciformis* haplotype (dA1/dA1), while the other four individuals H12, S1, S4 and S32 were homozygous for a *L. yunnanensis* haplotype (yA1/yA1).

**Table 3.** Haplotype combination for the putative hybrids of three nuclear loci and combined chloroplast fragments. UN means unique haplotype to the putative hybrids and haplotype names is in concordance with those in Fig. 2.

Locus	Haplotype combination	Number of individuals of the putative hybrids		Haplotype combination	Number of individuals of the putative hybrids	
		Type A	Type B		Type C	Type D
A12	cA1/dA1	14	8	dA1/yA1	26	27
	cA2/dA1	1	0	dA2/yA1	3	2
	cA1/UN1	0	1	dA1/dA1	0	2
	–	–	–	yA1/yA1	1	3
B14	cB1/dB1	1	3	dB1/yB1	18	19
	cB2/dB1	2	1	dB1/yB2	0	1
	cB3/dB1	6	2	dB1/UN7	0	1
	cB4/dB1	5	1	dB1/UN8	0	1
	dB1/UN2	0	1	dB1/UN9	1	0
	cB3/UN1	0	1	yB1/UN3	1	0
	dB1/dB1	1	0	yB1/UN4	0	1
	–	–	–	yB1/UN5	2	3
	–	–	–	UN5/UN6	0	1
	–	–	–	dB1/dB1	5	3
	–	–	–	yB1/yB1	1	4
	–	–	–	yB1/yB2	1	0
	–	–	–	yB2/yB2	1	0
D30	cD1/dD1	9	3	dD1/yD1	27	30
	cD2/dD1	1	0	dD1/dD1	1	2
	cD3/dD1	1	4	yD1/yD1	2	2
	cD4/dD1	1	0	–	–	–
	dD1/UN1	1	0	–	–	–
	dD1/UN2	1	2	–	–	–
	dD1/UN3	1	0	–	–	–
cpDNA	dP1	12	5	yP1	25	28
	cP1	0	2	dP1	2	3
	cP2	2	1	UN2	3	3
	cP3	0	1	–	–	–
	UN1	1	0	–	–	–

### Sequence analysis of nuclear loci—B14 locus

The length of aligned B14 fragment was 466 bp with one 2-bp indel distinguishing *L. duciformis* from *L. yunnanensis* (for variation sites, **see****Supporting Information—Table S2**). There were 21 haplotypes detected in total, of which 7 (cB1–7), 1 (dB1) and 4 (yB1–4) haplotypes were from *L. cyathiceps*, *L. duciformis* and *L. yunnanensis*, respectively (Table 2; Fig. 2B). Three clusters (cluster I, II and III) formed by haplotypes of *L. cyathiceps*, *L. duciformis* and *L. yunnanensis* were identified in haplotype network analysis, in which *L. cyathiceps* (cluster I) and *L. duciformis* (cluster II) were separated by six nucleotide substitutions and *L. duciformis* (cluster II) and *L. yunnanensis* (cluster III) were separated by three nucleotide substitutions (Fig. 2B).

For the putative hybrids of *L. cyathiceps* and *L. duciformis* (Type A and B), all individuals but one (F14) had two divergent haplotypes (cB1/dB1, cB2/dB1, cB3/dB1, cB4/dB1, dB1/UN2 and cB3/UN1) identified from *L. duciformis* and *L. cyathiceps* clusters, respectively (Table 3). Individual F14 is homozygous with the same haplotype of *L. duciformis* (dB1/dB1). Haplotypes for the putative hybrids of *L. duciformis* and *L. yunnanensis* (Type C and D) showed higher polymorphism with four types of haplotype composition as follows (Table 3): (i) The majority of individuals possessed two divergent haplotypes (dB1/yB1, dB1/yB2, dB1/UN7, dB1/UN8 and dB1/UN9), each nested in clusters of *L. duciformis* and *L. yunnanensis*, respectively. (ii) Eight individuals (H1, H4, H11, H13, H18, S16, S31 and S34) were homozygous for a *L. duciformis* haplotype (dB1/dB1). (iii) Six individuals (H16, H17, S1, S4, S21 and S32) were homozygous for one of *L. yunnanensis* haplotypes (yB1/yB1 and yB2/yB2). (iv) Nine individuals (H8, H12, H28, H30, S8, S11, S12, S14 and S33) showed mixed haplotypes (yB1/yB2, yB1/UN3, yB1/UN4, yB1/UN5 and UN5/UN6) originated from *L. yunnanensis* cluster.

### Sequence analysis of nuclear loci—D30 locus

The fragment D30 was 504 bp long after sequence alignment with one 1-bp indel distinguishing *L. cyathiceps* from *L. duciformis* and one 2-bp indel differing *L. duciformis* from *L. yunnanensis* (for variation sites, **see****Supporting Information—Table S3**). There were nine haplotypes identified in all individuals, of which four (cD1–4), one (dD1) and one (yD1) haplotypes were from *L. cyathiceps*, *L. duciformis* and *L. yunnanensis*, respectively (Table 2; Fig. 2C). In haplotype network analysis, three clusters (cluster I, II and III) were generated evidently by haplotypes of *L. cyathiceps*, *L. duciformis* and *L. yunnanensis*, respectively, in which *L. cyathiceps* (cluster I) and *L. duciformis* (cluster II) were separated by seven nucleotide substitutions and *L. duciformis* (cluster II) and *L. yunnanensis* (cluster III) were separated by 13 nucleotide substitutions (Fig. 2C).

For the putative hybrids of *L. cyathiceps* and *L. duciformis* (Type A and B), all individuals had two divergent haplotypes (cD1/dD1, cD2/dD1, cD3/dD1, cD4/dD1, dD1/UN1, dD1/UN2 and dD1/UN3) identified from *L. cyathiceps* and *L. duciformis* clusters, respectively (Table 3). For the putative hybrids of *L. duciformis* and *L. yunnanensis* (Type C and D), all individuals but seven (H12, H13, H26, S1, S8, S16 and S32) possessed combined haplotypes of *L. duciformis* and *L. yunnanensis* (dD1/yD1) (Table 3). Individuals H26, S8 and S16 were homozygous for the *L. duciformis* haplotype (dD1/dD1), whereas individuals H12, H13, S1 and S32 were homozygous for the *L. yunnanensis* haplotype (yD1/yD1).

### Sequence analysis of cpDNA fragments

The combined length of aligned cpDNA fragments (*psb*A–*trn*H, *trn*L–*rpl*32 and *trn*Q–5′*rps*16) was 2214 bp with 27 polymorphic sites and seven indels **[see****Supporting Information—Table S4****]**. Nine haplotypes were inferred in total, of which three (cP1–3), one (dP1) and three (yP1–3) haplotypes were from *L. cyathiceps*, *L. duciformis* and *L. yunnanensis*, respectively (Table 2; Fig. 2D). Haplotype network analysis indicated all three *L. cyathiceps* haplotypes (cP1–3) grouped into one cluster (cluster I), whereas two *L. yunnanensis* haplotypes (yP2–3) and the *L. duciformis* haplotype (dP1) formed into another cluster (cluster II) and another *L. yunnanensis* haplotype (yP1) was in the third cluster (cluster III) (Fig. 2D). Clusters I and II were separated by 23 nucleotide substitutions and clusters II and III were separated by 15 nucleotide substitutions.

For the putative hybrids of *L. cyathiceps* and *L. duciformis* (Type A and B), most (17 of 24) individuals had the same *L. duciformis* haplotype (dP1), and six individuals (F3, F11, T2, T3, T4 and T7) had haplotypes consistent with *L. cyathiceps* (cP1, cP2 and cP3) (Table 3). Individual F15 had a unique haplotype (UN1) with two nucleotide substitutions differed from the common *L. cyathiceps* haplotypes (cP1 and cP2). For the putative hybrids of *L. duciformis* and *L. yunnanensis* (Type C and D), most (53 of 64) individuals possessed haplotypes of *L. yunnanensis* (yP1) and five individuals (H3, H15, S6, S16 and S26) had haplotypes of *L. duciformis* (dP1) (Table 3). The other six individuals (H13, H22, H26, S24, S25 and S30) had a unique haplotype (UN2) differed from the haplotype of *L. yunnanensis* (yP1) with one mutation step. Genotypes at three low-copy nuclear loci and combined cpDNA fragments for all the investigated individuals are listed in **Supporting Information—Table S5**.

## Discussion

### Natural hybridization among *L. cyathiceps*, *L. duciformis* and *L. yunnanensis* from molecular evidence

In this study, we sequenced three low-copy nuclear loci and three cpDNA fragments to assess natural hybridization between *L. cyathiceps*, *L. duciformis* and *L. yunnanensis* in an area of contact in Tianchi, Shangri-La, Yunnan where the three species occur together. Our results suggested that the endemic *L. cyathiceps* showed relatively higher haplotype diversity than widely distributing *L. duciformis*, indicating high genetic diversity of *L. cyathiceps* at the study site. In addition, *L. duciformis* and *L. yunnanensis* showed closer genetic distance in two nuclear loci (A12 and B14) and combined cpDNA data, particularly in cpDNA data where two *L. yunnanensis* haplotypes grouped with the *L. duciformis* haplotype. These observations are consistent with the morphological classification (Liu 1989) and preliminary molecular phylogenetic results (W.-Y. He and Y.-Z. Pan, Kunming Institute of Botany, Chinese Academy of Sciences, unpubl. data). Nevertheless, *L. cyathiceps*, *L. duciformis* and *L. yunnanensis* remained well separated in the haplotype network analysis, indicating their clear divergence from each other. In general, morphologically intermediate individuals Type A and B showed chromatogram additivity for *L. cyathiceps* and *L. duciformis* at most nuclear loci, while most Type C and D individuals for *L. duciformis* and *L. yunnanensis*, providing strong evidence for natural hybridization hypotheses above and for lack of hybridization between *L. cyathiceps* and *L. yunnanensis*. Additionally, the occurrence of unique haplotypes in putative hybrids of two hybridization groups may be intragenic recombination between haplotypes or caused by unsampled polymorphisms in parental individuals.

### Pre- and post-zygotic barriers among *L. cyathiceps*, *L. duciformis* and *L. yunnanensis*

Different pre- and post-zygotic barriers can reduce potential cross-breeding and result in reproductive isolation between species pairs (Dobzhansky 1937; Grant 1981; Stace 1991; Field *et al.* 2011; Ma *et al.* 2016). Meanwhile, natural hybridization may occur between closely related species with incomplete pre- and post-zygotic barriers. Natural hybridization is often associated with disturbed habitats as human disturbance can disrupt ecological barriers and promote natural hybridization (Anderson 1948; Rieseberg and Carney 1998; Ma *et al.* 2010). Human disturbance can create intermediate habitat suitable for hybrids, promoting the maintenance of hybrid swarms in these habitats (Anderson 1948; Ellstrand and Schierenbeck 2000). In previous hybridization studies of *Ligularia*, hybrid zones often locate at roadsides, mountain slopes destroyed by fire and other areas subjected to human disturbance, suggesting that hybridization in *Ligularia* may be promoted by human activities (Pan *et al.* 2008; Yu *et al.* 2011, 2014a, b). In this study, once again, the hybrid zone is located in an area severely disturbed by human activities such as tree felling and grazing, supporting the observation of previous studies.

But, could pre- and post-zygotic barriers explain the hybridization patterns observed in this contact zone? The overlap of blooming periods provides the first condition for hybridization since it enables pollen movement by pollinator vectors. In the present study, both *L. duciformis* and *L. cyathiceps* flower from July to August, while *L. yunnanensis* flowers from May to August (Liu and Illarionova 2011). Moreover, *Ligularia* plants have generalized pollination system and about 10 insects belonging to three orders (Diptera, Lepidoptera and Hymenoptera) are the major pollinators (Liu 2002; Cao *et al.* 2008). Generalized pollinators shared between species offer opportunities for the pollen transfer. Thus, incomplete pre-zygotic barriers such as the overlap of blooming periods and generalized pollinators will largely contribute for natural hybridization of *L. cyathiceps* × *L. duciformis* and *L. duciformis* × *L. yunnanensis*.

Similar inflorescence arrangement may be another factor significantly contributing for the hybridization between *L. duciformis* and *L. yunnanensis*. These two species present similar arrangement of the capitula in corymb inflorescences. The generalized pollinators may tend to visit inflorescences with similar morphologies, thus promoting pollen transfer between these two species. Indeed, pollen transfer between species with similar inflorescence arrangement has been observed in natural hybrid zones of *Ligularia* (Pan *et al.* 2008; Yu *et al.* 2014b).

In addition, close relationship between *L. duciformis* and *L. yunnanensis* may also work as a less effective post-zygotic barrier to hybridization in closely related species. In previous studies, hybridization has been detected in *Ligularia* species pairs that are closely related, such as *L. paradoxa* and *L. duciformis* (Pan *et al.* 2008) and *L. cymbulifera* and *L. tongolensis* (Yu *et al.* 2014b). In the present study, *L. duciformis* and *L. yunnanensis* both belong to Series *Retusae*, Section *Corymbosae* (Liu 1989) and are closely related according to haplotype analysis (Results section in this study) and preliminary molecular phylogenetic study (W.-Y. He and Y.-Z. Pan, Kunming Institute of Botany, Chinese Academy of Sciences, unpubl. data).

Since *L. cyathiceps* and *L. yunnanensis* also possess the overlapping blooming periods and generalized pollinators, it seems that there are no pre-zygotic barriers reducing pollen transfer between them. The lack of hybrids between *L. cyathiceps* and *L. yunnanensis* may be attributed to post-zygotic barriers. Actually, sympatric species in *Ligularia* could coexist without hybridization, if they have undergone long isolation and accumulated enough mutations, as indicated by species in the *Ligularia*-*Cremanthodium*-*Parasenecio* (*L-C-P*) complex (Liu *et al.* 2006). In the network analysis of three low-copy nuclear loci and combined cpDNA fragments, *L. cyathiceps* and *L. yunnanensis* showed relatively higher genetic distance than each of them with *L. duciformis*. Although the genetic difference shown in the network analysis is limited, it may be caused by the restricted loci used in this study. Therefore, the accumulation of mutations between *L. cyathiceps* and *L. yunnanensis* may drive the species divergence, reduce interspecies crossability and/or lower fitness of possible hybrids. Post-zygotic barriers resulting in the aborted seeds or reduction of fitness for hybrids in seedling stages, which have been observed in many plants, such as *Chamaecrista* (Costa *et al.* 2007), *Rhododendron* (Ma *et al.* 2016) and *Silene* (Zhang *et al.* 2016), may lead to the lack of hybrids between *L. cyathiceps* and *L. yunnanensis*.

### Asymmetric hybridization of *L. cyathiceps* × *L. duciformis* and *L. duciformis* × *L. yunnanensis*

As *Ligularia* was proved to be chloroplast maternally inherited (Zhang *et al.* 2003), combined cpDNA fragments would predict the direction of natural hybridization. For the hybridization *L. cyathiceps* × *L. duciformis*, cpDNA data indicate *L. duciformis* is the maternal parent of most (70.83 %) putative hybrids, thus natural hybridization between *L. cyathiceps* and *L. duciformis* is bidirectional but asymmetric, and *L. duciformis* is the primary maternal parent. However, for the hybridization *L. duciformis* × *L. yunnanensis*, cpDNA results suggest that *L. yunnanensis* is the maternal parent of most (82.81 %) putative hybrids. Two hybridization groups show distinctive asymmetry in natural hybridization and different factors may be responsible for their asymmetry.

Differences in floral traits could drive differences in floral preferences and floral constancy of pollinators, which may affect the levels and direction of hybridization (Aldridge and Campbell 2007; Castro *et al.* 2011). This could be occurring, for example, between *L. duciformis* and *L. cyathiceps*. *Ligularia duciformis* have larger compound corymb inflorescences than the racemose inflorescences of *L. cyathiceps*; therefore, it would be likely that *L. duciformis* is more attractive to pollinators and acts as the maternal parent to accept pollen transferred from *L. cyathiceps*.

Contrarily, for *L. duciformis* and *L. yunnanensis*, having similar inflorescence traits, the asymmetric hybridization may be associated to the relative abundance of parental species. The prediction that the rare species, undergoing ‘pollen swamping’ by more abundant congeners, usually acts as the maternal parent, is confirmed by many examples in plants and animals (Arnold *et al.* 1993; Rieseberg 1995; Levin *et al.* 1996; Wirtz 1999; Lepais *et al.* 2009). At the present study site, *L. duciformis* occupies more widely habitat than *L. yunnanensis* occurring in intact habitat, and *L. duciformis* plants greatly outnumber *L. yunnanensis* plants, thus *L. yunnanensis* would be more likely the maternal parent.

### Consequences of natural hybridization among *L. cyathiceps*, *L. duciformis* and *L. yunnanensis*

In the present study, most putative hybrid individuals in two hybridization groups show chromatogram additivity at all of three randomly selected nuclear loci, suggesting they might be F_1_s. Hybrids restricted to F_1_ generation can impede gene flow between species and keep hybridizing species pairs reproductively isolated from each other (Milne *et al.* 2003; Milne and Abbott 2008; Twyford *et al.* 2015). Four morphologically intermediate individuals S16 and H12, S1, S32 are pure with haplotypes of *L. duciformis* and *L. yunnanensis*, respectively, at three nuclear loci. It might be unlikely that these homozygous individuals are caused by repetitive backcrossing with corresponding parents, since there is no occurrence of later-generation individuals. They may result from sampling confusion mistakes between hybrids and pure parents, indicating further morphological studies are needed in this area of contact. There are two morphology-differential types of hybrids produced by two hybridization groups, especially in the *L. cyathiceps* and *L. duciformis* hybridization group where Type A and Type B differ in the presence/absence of ray floret. Nevertheless, it is noteworthy that different types in these two hybridization groups show similar intra-group nuclear and chloroplast haplotype composition.

In previous reports on natural hybridization of *Ligularia*, hybrid swarms are common and introgression occurs between parental species (Yu *et al.* 2011, 2014a, b). Being a genus with high species diversity formed by rapid radiation (Liu *et al.* 2006), species in *Ligularia* may not be completely isolated reproductively and sympatric hybridization is expected to be frequent. However, the existence of F_1_ hybrids without later-generation individuals prevents introgression and facilitates reproductive isolation among sympatric species. Moreover, the lack of later-generation hybrids seems to be the result of a fitness disadvantage of the hybrids produced by *L. cyathiceps* × *L. duciformis* and *L. duciformis* × *L. yunnanensis*, which is also a barrier to hybridization at a more advanced stage. Unlike the F_1_-dominated hybrid zone in *Rhododendron* descripted by Milne *et al.* (2003), hybridization in this study might be promoted by human disturbance such as tree felling and grazing; however, post-zygotic barriers such as the sterility of F_1_s may contribute for no later-generation hybrids. Human disturbance can bring species into contact and trigger natural hybridization (Anderson 1948), and can furtherly promote hybridization through increasing opportunities for gene flow (Lamont *et al.* 2003; Zha *et al.* 2009; Thompson *et al.* 2010; Ortego *et al.* 2017). In the present study, although human disturbance might influence or promote the hybridization among the three *Ligularia* species studied, they may still keep their species distinctiveness and maintain reproductive isolation under the circumstance that hybridization takes place. Nevertheless, in the future studies, experiments such as controlled pollination crosses, seed germination and hybrid fitness examination need to be conducted to furtherly reveal the asymmetric hybridization and pre- and post-zygotic isolating barriers among three *Ligularia* species in this hybrid zone in the hotspot area of Northwest Yunnan.

## Conclusions

The natural hybridization of *L. cyathiceps* × *L. duciformis* and *L. duciformis* × *L. yunnanensis* was confirmed based on three low-copy nuclear loci and three cpDNA fragments. In the two hybridization groups, most hybrids seem to be F_1_s, which suggests the maintenance of species boundaries between hybridizing species. There were no hybrids between *L. cyathiceps* and *L. yunnanensis*, which may be attributed to post-zygotic reproductive barriers such as hybrid inviability and sterility. Chloroplast DNA data indicated asymmetric hybridization, with *L. duciformis* as primary maternal parent in the *L. cyathiceps* × *L. duciformis* hybridization group, and *L. yunnanensis* for the *L. duciformis* × *L. yunnanensis* hybridization group. Pollinator preferences and the relative abundance of parental species may lead to asymmetric hybridization. Still, the three *Ligularia* species seem to maintain the species integrity in the studied sympatric area.

## Accession Numbers

The data set of DNA sequencing data have been deposited in GenBank under accession numbers KX779147–KX779271.

## Sources of Funding

This research was supported by the National Natural Science Foundation of China (grant no. 31600178 to J.-J.Y.).

## Contributions by the Authors

X.G. and Y.-H.W. conceived and designed the experiments. X.G. and J.-J.Y. collected plant materials. N.-N.Z. performed the experiments, analysed the data and wrote the manuscript. X.G. and J.-J.Y. revised the manuscript. All authors read and approved the final manuscript.

## Conflict of Interest

None declared.

## Supporting Information

The following additional information is available in the online version of this article—

**Table S1**. Variation sites of A12 locus for six haplotypes in all investigated individuals.

**Table S2**. Variation sites and indels of B14 locus for 21 haplotypes in all investigated individuals.

**Table S3**. Variation sites and indels of D30 locus for nine haplotypes in all investigated individuals.

**Table S4**. Variation sites and indels of three chloroplast fragments for nine haplotypes in all investigated individuals.

**Table S5**. Genotypes at three low-copy nuclear loci and combined cpDNA fragments for all the investigated individuals.

## Supplementary Material

Supporting Information
