# Supplementary material for: Molecular evidence for asymmetric hybridization in three closely related sympatric species
Source: AoB Plants. 2018 Feb 9;10(1):ply011. doi: 10.1093/aobpla/ply011 (PMC5824843; doi:10.1093/aobpla/ply011)
Supplement: Supporting Information [file ply011_suppl_supporting_information.docx]

**Table S1.** Variation sites of A12 locus for six haplotypes in all investigated individuals.

| Haplotype | Nucleotide position | | | | | | | | | | | | |
| --- | --- | --- | --- | --- | --- | --- | --- | --- | --- | --- | --- | --- | --- |
|  | 23-24 | 47 | 50 | 62 | 71 | 113 | 123 | 131 | 133 | 135 | 138 | 147 | 243 |
| cA1 | GT | T | G | A | C | C | C | C | T | T | G | A | C |
| cA2 | GT | T | G | A | C | C | C | C | A | T | G | A | C |
| dA1 | GT | C | G | T | T | C | A | T | T | T | T | C | C |
| dA2 | GT | C | G | A | T | C | A | T | T | T | T | C | C |
| yA1 | GT | C | T | A | C | C | C | T | T | A | G | C | C |
| UN | CA | C | G | A | C | A | C | T | T | T | G | A | T |

**Table S2.** Variation sites and indels of B14 locus for twenty-one haplotypes in all investigated individuals.

| Haplotype | Nucleotide position | | | | | | | | | | | | | | | | | | | | | | | | | | | | |
| --- | --- | --- | --- | --- | --- | --- | --- | --- | --- | --- | --- | --- | --- | --- | --- | --- | --- | --- | --- | --- | --- | --- | --- | --- | --- | --- | --- | --- | --- |
|  | 38 | 56 | 60 | 63 | 68 | 83 | 89-90 | 94 | 102 | 121 | 124 | 183 | 197 | 199 | 215 | 218 | 227 | 256 | 262 | 281 | 286 | 300 | 321 | 333 | 339 | 343 | 402 | 405 | 458 |
| cB1 | T | A | T | C | C | A | AA | C | A | G | G | T | A | C | T | T | A | G | C | G | G | A | C | G | C | G | T | A | G |
| cB2 | T | A | T | C | C | A | AA | C | A | G | G | T | A | C | T | T | A | A | C | G | G | A | C | G | C | G | T | G | G |
| cB3 | T | A | T | C | C | A | AA | C | A | G | G | T | A | C | T | T | A | G | C | G | G | A | C | G | C | G | T | G | G |
| cB4 | C | A | T | C | C | A | AA | C | A | G | A | T | A | C | T | T | A | G | C | G | G | A | C | G | C | G | T | G | G |
| cB5 | T | A | T | C | C | A | AA | C | A | C | G | T | A | C | T | A | A | G | T | G | G | A | C | T | C | G | T | G | G |
| cB6 | C | A | T | C | C | A | AA | C | A | G | A | T | A | C | T | T | A | A | C | G | G | A | C | G | C | G | T | G | G |
| cB7 | T | A | T | C | C | A | AA | C | A | G | G | T | A | C | T | T | A | A | C | G | G | A | C | G | C | A | T | G | G |
| dB1 | C | A | T | C | A | A | AA | A | A | G | G | C | A | C | A | T | C | G | C | G | G | A | C | G | C | C | T | G | T |
| yB1 | C | T | T | C | C | A | — | C | G | G | G | C | A | C | T | T | C | G | C | G | G | G | T | G | C | C | T | G | T |
| yB2 | C | T | T | C | C | A | — | C | A | G | G | C | A | C | T | T | C | G | C | G | G | G | T | G | C | C | T | G | T |
| yB3 | C | A | T | C | C | T | — | C | G | G | G | C | A | C | T | T | C | G | C | A | A | G | T | G | C | C | T | G | T |
| yB4 | C | A | T | C | C | T | — | C | G | G | G | C | G | C | T | T | C | G | C | A | A | G | T | G | C | C | T | G | T |
| UN1 | T | A | T | C | A | A | AA | A | A | G | G | C | A | C | A | T | C | G | C | G | G | A | C | G | C | C | T | G | G |
| UN2 | C | A | T | C | C | A | AA | C | A | G | A | T | A | A | T | T | A | G | C | G | G | A | C | G | C | G | T | G | G |
| UN3 | C | T | T | C | C | A | — | C | G | G | G | C | A | C | T | T | C | G | C | G | G | G | C | G | C | C | T | G | T |
| UN4 | C | T | T | T | C | A | — | C | G | G | G | C | A | C | T | T | C | G | C | G | G | G | T | G | C | C | T | G | T |
| UN5 | C | A | T | T | C | A | — | C | G | G | G | C | A | C | T | T | C | G | C | G | G | G | T | G | T | C | T | G | T |
| UN6 | C | T | T | C | C | A | AA | A | A | G | G | C | A | C | A | T | C | G | C | G | G | A | C | G | C | C | T | G | T |
| UN7 | C | T | T | C | C | A | — | C | G | G | G | C | A | C | T | T | C | G | C | G | G | A | T | G | C | C | T | G | T |
| UN8 | C | T | T | C | C | A | — | C | G | G | G | C | A | C | T | T | C | G | C | G | G | G | T | G | C | C | G | G | T |
| UN9 | C | T | C | C | C | A | — | C | G | G | G | C | A | C | T | T | C | G | C | G | G | G | T | G | C | C | T | G | T |

Note: “—” means nucleotide deletion.

**Table S3.** Variation sites and indels of D30 locus for nine haplotypes in all investigated individuals.

| Haplotype | Nucleotide position | | | | | | | | | | | | | | | | | | |
| --- | --- | --- | --- | --- | --- | --- | --- | --- | --- | --- | --- | --- | --- | --- | --- | --- | --- | --- | --- |
|  | 6 | 35 | 47 | 185-186 | 192-193 | 200 | 222 | 230 | 251 | 265 | 298 | 314 | 320 | 328 | 331 | 332 | 346 | 413 | 480 |
| cD1 | C | G | A | AT | TT | C | T | G | G | G | G | A | T | T | G | A | T | T | A |
| cD2 | C | G | A | AT | TT | C | — | G | G | G | G | A | T | T | A | A | C | T | A |
| cD3 | C | G | A | AT | TT | C | T | G | G | G | G | A | T | T | G | A | C | T | A |
| cD4 | C | G | A | AT | TT | C | — | G | G | G | G | A | T | T | G | A | C | T | A |
| dD1 | G | T | A | GA | TT | T | T | G | G | G | A | C | C | T | G | A | C | G | A |
| yD1 | C | G | G | AT | — | C | T | A | A | A | G | C | C | A | G | G | C | G | G |
| UN1 | C | G | A | AT | TT | C | — | G | G | G | G | A | T | T | G | A | T | T | A |
| UN2 | C | G | A | AT | TT | C | T | G | G | G | G | A | T | T | A | A | C | T | A |
| UN3 | C | G | A | AT | TT | C | — | G | G | G | G | A | T | T | G | A | C | G | A |

Note: “—” means nucleotide deletion.

**Table S4.** Variation sites and indels of three chloroplast fragments for nine haplotypes in all investigated individuals.

|  | *psb*A-*trn*H | | | |  | *trn*L-*rpl*32 | | | | | | | | | | | | | | | |  | *trn*Q-5’*rps*16 | | | | | | | | | | | | | |
| --- | --- | --- | --- | --- | --- | --- | --- | --- | --- | --- | --- | --- | --- | --- | --- | --- | --- | --- | --- | --- | --- | --- | --- | --- | --- | --- | --- | --- | --- | --- | --- | --- | --- | --- | --- | --- |
| Haplotype | 13  ~  17 | 134 | 136~  137 | 396 |  | 5 | 124~  127 | 131 | 188~  195 | 230 | 251~  252 | 269 | 329 | 335  ~  337 | 340 | 366 | 455~  456 | 499 | 504~  509 | 745~  754 | 843 |  | 9 | 82  ~  93 | 160 | 268 | 381 | 394 | 498~  509 | 519 | 522 | 586 | 746 | 798 | 857 | 884 |
| cP1 | ① | T | TT | G |  | T | — | T | ③ | C | AA | C | A | TTT | C | G | AT | T | — | — | A |  | T | — | G | A | C | G | — | A | A | A | A | G | A | G |
| cP2 | — | T | TT | G |  | T | — | T | ③ | C | AA | C | A | TTT | C | G | AT | T | ④ | — | A |  | T | — | G | A | C | G | — | A | A | A | A | G | A | G |
| cP3 | — | T | TT | G |  | T | — | T | ③ | C | AA | C | A | TTT | C | T | AT | T | — | — | A |  | T | — | G | A | C | G | — | A | A | A | G | G | A | G |
| dP1 | — | A | AA | G |  | A | ② | G | — | A | AT | C | A | TTT | A | G | CA | T | — | ⑤ | G |  | A | — | T | C | C | C | — | C | T | C | A | T | T | C |
| yP1 | — | T | TT | G |  | A | — | T | ③ | A | TT | C | A | AAA | C | G | AT | C | — | ⑤ | G |  | A | ⑥ | T | A | A | C | ⑦ | C | T | C | A | G | A | C |
| yP2 | — | A | AA | G |  | A | ② | G | — | A | TT | T | C | ATT | A | G | CA | T | — | ⑤ | G |  | A | — | T | C | C | C | — | C | T | C | A | T | T | C |
| yP3 | — | A | AA | A |  | A | ② | G | — | A | TT | T | C | ATT | A | G | CA | T | — | ⑤ | G |  | A | — | T | C | C | C | — | C | T | C | A | T | T | C |
| UN1 | — | T | TT | G |  | T | — | T | — | C | AA | C | A | TTT | C | G | AT | T | — | — | A |  | T | — | G | A | C | G | — | A | A | A | A | G | A | G |
| UN2 | — | T | TT | A |  | A | — | T | ③ | A | TT | C | A | AAA | C | G | AT | C | — | ⑤ | G |  | A | ⑥ | T | A | A | C | — | C | T | C | A | G | A | C |

Note: “—” means nucleotide deletion, and numbers ① to ⑦ represent inserted nucleotides as follows: ①: TAGTA; ②: TTTC; ③: ATTATAAG; ④: TATTAT; ⑤: AAGGAAAATA; ⑥: TAGATAATAAAA; ⑦: TAGATCGAACAA.

**Table S5.** Genotypes at three low-copy nuclear loci and combined cpDNA fragments for all the investigated individuals.

| Species | Individual No. | A12 | B14 | D30 | cpDNA |
| --- | --- | --- | --- | --- | --- |
| *L. cyathiceps* | C1 | cA1/cA1 | cB3/cB7 | cD1/cD1 | cP1 |
|  | C2 | cA1/cA1 | cB2/cB6 | cD1/cD1 | cP2 |
|  | C3 | cA1/cA1 | cB4/cB6 | cD1/cD1 | cP1 |
|  | C4 | cA1/cA1 | cB3/cB4 | cD1/cD1 | cP1 |
|  | C5 | cA1/cA1 | cB4/cB4 | cD3/cD3 | cP1 |
|  | C6 | cA1/cA2 | cB3/cB4 | cD1/cD1 | cP2 |
|  | C7 | cA1/cA1 | cB4/cB4 | cD4/cD4 | cP3 |
|  | C8 | cA1/cA1 | cB3/cB3 | cD2/cD2 | cP1 |
|  | C9 | cA1/cA1 | cB1/cB4 | cD1/cD1 | cP2 |
|  | C10 | cA1/cA1 | cB3/cB4 | cD1/cD3 | cP2 |
|  | C11 | cA1/cA2 | cB2/cB3 | cD1/cD1 | cP2 |
|  | C12 | cA1/cA1 | cB1/cB1 | cD1/cD1 | cP2 |
|  | C13 | cA1/cA1 | cB4/cB4 | cD1/cD1 | cP1 |
|  | C14 | cA1/cA1 | cB3/cB3 | cD1/cD1 | cP1 |
|  | C15 | cA1/cA1 | cB4/cB4 | cD1/cD1 | cP1 |
|  | C16 | cA1/cA1 | cB4/cB4 | cD1/cD1 | cP1 |
|  | C17 | cA1/cA1 | cB4/cB5 | cD1/cD1 | cP1 |
|  | C18 | cA1/cA2 | cB3/cB4 | cD1/cD1 | cP1 |
|  | C19 | cA1/cA1 | cB3/cB3 | cD1/cD1 | cP3 |
|  | C20 | cA1/cA1 | cB3/cB3 | cD1/cD3 | cP1 |
| Type A | F1 | dA1/cA1 | dB1/cB4 | dD1/cD1 | dP1 |
|  | F2 | dA1/cA2 | dB1/cB2 | dD1/cD1 | dP1 |
|  | F3 | dA1/cA1 | dB1/cB3 | dD1/cD1 | cP2 |
|  | F4 | dA1/cA1 | dB1/cB4 | dD1/cD1 | dP1 |
|  | F5 | dA1/cA1 | dB1/cB4 | dD1/cD2 | dP1 |
|  | F6 | dA1/cA1 | dB1/cB4 | dD1/cD4 | dP1 |
|  | F7 | dA1/cA1 | dB1/cB3 | dD1/UN3 | dP1 |
|  | F8 | dA1/cA1 | dB1/cB1 | dD1/cD1 | dP1 |
|  | F9 | dA1/cA1 | dB1/cB3 | dD1/UN2 | dP1 |
|  | F10 | dA1/cA1 | dB1/cB3 | dD1/cD3 | dP1 |
|  | F11 | dA1/cA1 | dB1/cB3 | dD1/cD1 | cP2 |
|  | F12 | dA1/cA1 | dB1/cB2 | dD1/UN1 | dP1 |
|  | F13 | dA1/cA1 | dB1/cB4 | dD1/cD1 | dP1 |
|  | F14 | dA1/cA1 | dB1/dB1 | dD1/cD1 | dP1 |
|  | F15 | dA1/cA1 | dB1/cB3 | dD1/cD1 | UN1 |
| Type B | T1 | dA1/cA1 | dB1/cB1 | dD1/cD3 | dP1 |
|  | T2 | dA1/cA1 | dB1/cB3 | dD1/UN2 | cP1 |
|  | T3 | dA1/cA1 | UN1/cB3 | dD1/cD3 | cP2 |
|  | T4 | dA1/cA1 | dB1/cB1 | dD1/cD1 | cP1 |
|  | T5 | dA1/cA1 | dB1/cB4 | dD1/UN2 | dP1 |
|  | T6 | dA1/cA1 | dB1/UN2 | dD1/cD3 | dP1 |
|  | T7 | cA1/UN1 | dB1/cB1 | dD1/cD1 | cP3 |
|  | T8 | dA1/cA1 | dB1/cB3 | dD1/cD1 | dP1 |
|  | T9 | dA1/cA1 | dB1/cB2 | dD1/cD3 | dP1 |
| *L. duciformis* | D1 | dA1/dA1 | dB1/dB1 | dD1/dD1 | dP1 |
|  | D2 | dA1/dA1 | dB1/dB1 | dD1/dD1 | dP1 |
|  | D3 | dA1/dA1 | dB1/dB1 | dD1/dD1 | dP1 |
|  | D4 | dA1/dA1 | dB1/dB1 | dD1/dD1 | dP1 |
|  | D5 | dA1/dA1 | dB1/dB1 | dD1/dD1 | dP1 |
|  | D6 | dA1/dA1 | dB1/dB1 | dD1/dD1 | dP1 |
|  | D7 | dA2/dA2 | dB1/dB1 | dD1/dD1 | dP1 |
|  | D8 | dA1/dA1 | dB1/dB1 | dD1/dD1 | dP1 |
|  | D9 | dA1/dA1 | dB1/dB1 | dD1/dD1 | dP1 |
|  | D10 | dA1/dA1 | dB1/dB1 | dD1/dD1 | dP1 |
|  | D11 | dA1/dA1 | dB1/dB1 | dD1/dD1 | dP1 |
|  | D12 | dA1/dA1 | dB1/dB1 | dD1/dD1 | dP1 |
|  | D13 | dA1/dA1 | dB1/dB1 | dD1/dD1 | dP1 |
|  | D14 | dA1/dA1 | dB1/dB1 | dD1/dD1 | dP1 |
|  | D15 | dA1/dA1 | dB1/dB1 | dD1/dD1 | dP1 |
|  | D16 | dA1/dA1 | dB1/dB1 | dD1/dD1 | dP1 |
|  | D17 | dA1/dA1 | dB1/dB1 | dD1/dD1 | dP1 |
|  | D18 | dA1/dA1 | dB1/dB1 | dD1/dD1 | dP1 |
|  | D19 | dA1/dA1 | dB1/dB1 | dD1/dD1 | dP1 |
|  | D20 | dA1/dA1 | dB1/dB1 | dD1/dD1 | dP1 |
| Type C | H1 | dA1/yA1 | dB1/dB1 | dD1/yD1 | yP1 |
|  | H2 | dA1/yA1 | dB1/yB1 | dD1/yD1 | yP1 |
|  | H3 | dA1/yA1 | dB1/yB1 | dD1/yD1 | dP1 |
|  | H4 | dA1/yA1 | dB1/dB1 | dD1/yD1 | yP1 |
|  | H5 | dA2/yA1 | dB1/yB1 | dD1/yD1 | yP1 |
|  | H6 | dA1/yA1 | dB1/yB1 | dD1/yD1 | yP1 |
|  | H7 | dA1/yA1 | dB1/yB1 | dD1/yD1 | yP1 |
|  | H8 | dA1/yA1 | UN5/yB1 | dD1/yD1 | yP1 |
|  | H9 | dA1/yA1 | dB1/yB1 | dD1/yD1 | yP1 |
|  | H10 | dA1/yA1 | dB1/yB1 | dD1/yD1 | yP1 |
|  | H11 | dA1/yA1 | dB1/dB1 | dD1/yD1 | yP1 |
|  | H12 | yA1/yA1 | yB2/yB1 | yD1/yD1 | yP1 |
|  | H13 | dA1/yA1 | dB1/dB1 | yD1/yD1 | UN2 |
|  | H14 | dA1/yA1 | dB1/yB1 | dD1/yD1 | yP1 |
|  | H15 | dA1/yA1 | dB1/yB1 | dD1/yD1 | dP1 |
|  | H16 | dA1/yA1 | yB1/yB1 | dD1/yD1 | yP1 |
|  | H17 | dA2/yA1 | yB2/yB2 | dD1/yD1 | yP1 |
|  | H18 | dA1/yA1 | dB1/dB1 | dD1/yD1 | yP1 |
|  | H19 | dA1/yA1 | dB1/yB1 | dD1/yD1 | yP1 |
|  | H20 | dA1/yA1 | dB1/yB1 | dD1/yD1 | yP1 |
|  | H21 | dA1/yA1 | dB1/yB1 | dD1/yD1 | yP1 |
|  | H22 | dA1/yA1 | dB1/yB1 | dD1/yD1 | UN2 |
|  | H23 | dA1/yA1 | dB1/yB1 | dD1/yD1 | yP1 |
|  | H24 | dA1/yA1 | dB1/yB1 | dD1/yD1 | yP1 |
|  | H25 | dA1/yA1 | dB1/yB1 | dD1/yD1 | yP1 |
|  | H26 | dA1/yA1 | dB1/yB1 | dD1/dD1 | UN2 |
|  | H27 | dA2/yA1 | dB1/UN9 | dD1/yD1 | yP1 |
|  | H28 | dA1/yA1 | UN5/yB1 | dD1/yD1 | yP1 |
|  | H29 | dA1/yA1 | dB1/yB1 | dD1/yD1 | yP1 |
|  | H30 | dA1/yA1 | UN3/yB1 | dD1/yD1 | yP1 |
| Type D | S1 | yA1/yA1 | yB1/yB1 | yD1/yD1 | yP1 |
|  | S2 | dA1/yA1 | dB1/yB1 | dD1/yD1 | yP1 |
|  | S3 | dA1/yA1 | dB1/yB1 | dD1/yD1 | yP1 |
|  | S4 | yA1/yA1 | yB1/yB1 | dD1/yD1 | yP1 |
|  | S5 | dA1/yA1 | dB1/yB1 | dD1/yD1 | yP1 |
|  | S6 | dA1/yA1 | dB1/yB1 | dD1/yD1 | dP1 |
|  | S7 | dA1/yA1 | dB1/yB1 | dD1/yD1 | yP1 |
|  | S8 | dA1/dA1 | UN5/yB1 | dD1/dD1 | yP1 |
|  | S9 | dA1/yA1 | dB1/yB1 | dD1/yD1 | yP1 |
|  | S10 | dA2/yA1 | dB1/yB1 | dD1/yD1 | yP1 |
|  | S11 | dA1/yA1 | UN5/yB1 | dD1/yD1 | yP1 |
|  | S12 | dA1/yA1 | UN5/yB1 | dD1/yD1 | yP1 |
|  | S13 | dA1/yA1 | dB1/UN7 | dD1/yD1 | yP1 |
|  | S14 | dA1/yA1 | UN5/UN6 | dD1/yD1 | yP1 |
|  | S15 | dA1/yA1 | dB1/yB1 | dD1/yD1 | yP1 |
|  | S16 | dA1/dA1 | dB1/dB1 | dD1/dD1 | dP1 |
|  | S17 | dA1/yA1 | dB1/yB1 | dD1/yD1 | yP1 |
|  | S18 | dA1/yA1 | dB1/yB1 | dD1/yD1 | yP1 |
|  | S19 | dA1/yA1 | dB1/yB1 | dD1/yD1 | yP1 |
|  | S20 | dA1/yA1 | dB1/yB1 | dD1/yD1 | yP1 |
|  | S21 | dA1/yA1 | yB1/yB1 | dD1/yD1 | yP1 |
|  | S22 | dA2/yA1 | dB1/UN8 | dD1/yD1 | yP1 |
|  | S23 | dA1/yA1 | dB1/yB1 | dD1/yD1 | yP1 |
|  | S24 | dA1/yA1 | dB1/yB1 | dD1/yD1 | UN2 |
|  | S25 | dA1/yA1 | dB1/yB1 | dD1/yD1 | UN2 |
|  | S26 | dA1/yA1 | dB1/yB2 | dD1/yD1 | dP1 |
|  | S27 | dA1/yA1 | dB1/yB1 | dD1/yD1 | yP1 |
|  | S28 | dA1/yA1 | dB1/yB1 | dD1/yD1 | yP1 |
|  | S29 | dA1/yA1 | dB1/yB1 | dD1/yD1 | yP1 |
|  | S30 | dA1/yA1 | dB1/yB1 | dD1/yD1 | UN2 |
|  | S31 | dA1/yA1 | dB1/dB1 | dD1/yD1 | yP1 |
|  | S32 | yA1/yA1 | yB1/yB1 | yD1/yD1 | yP1 |
|  | S33 | dA1/yA1 | yB1/UN4 | dD1/yD1 | yP1 |
|  | S34 | dA1/yA1 | dB1/dB1 | dD1/yD1 | yP1 |
| *L. yunnanensis* | Y1 | yA1/yA1 | yB1/yB1 | yD1/yD1 | yP2 |
|  | Y2 | yA1/yA1 | yB1/yB1 | yD1/yD1 | yP1 |
|  | Y3 | yA1/yA1 | yB1/yB1 | yD1/yD1 | yP1 |
|  | Y4 | yA1/yA1 | yB1/yB1 | yD1/yD1 | yP3 |
|  | Y5 | yA1/yA1 | yB1/yB2 | yD1/yD1 | yP1 |
|  | Y6 | yA1/yA1 | yB1/yB2 | yD1/yD1 | yP1 |
|  | Y7 | yA1/yA1 | yB1/yB1 | yD1/yD1 | yP1 |
|  | Y8 | yA1/yA1 | yB1/yB1 | yD1/yD1 | yP2 |
|  | Y9 | yA1/yA1 | yB1/yB1 | yD1/yD1 | yP1 |
|  | Y10 | yA1/yA1 | yB4/yB4 | yD1/yD1 | yP2 |
|  | Y11 | yA1/yA1 | yB1/yB1 | yD1/yD1 | yP2 |
|  | Y12 | yA1/yA1 | yB1/yB2 | yD1/yD1 | yP3 |
|  | Y13 | yA1/yA1 | yB1/yB1 | yD1/yD1 | yP2 |
|  | Y14 | yA1/yA1 | yB1/yB2 | yD1/yD1 | yP1 |
|  | Y15 | yA1/yA1 | yB3/yB3 | yD1/yD1 | yP2 |
|  | Y16 | yA1/yA1 | yB2/yB2 | yD1/yD1 | yP3 |
|  | Y17 | yA1/yA1 | yB2/yB2 | yD1/yD1 | yP1 |
|  | Y18 | yA1/yA1 | yB1/yB1 | yD1/yD1 | yP2 |
|  | Y19 | yA1/yA1 | yB1/yB1 | yD1/yD1 | yP2 |
|  | Y20 | yA1/yA1 | yB2/yB2 | yD1/yD1 | yP2 |
